# Transitions between degrees of multidimensional frailty among older people admitted to intermediate care: a multicentre prospective study

**DOI:** 10.1186/s12877-022-03378-9

**Published:** 2022-09-01

**Authors:** Jordi Amblàs-Novellas, Anna Torné, Ramon Oller, Joan Carles Martori, Joan Espaulella, Roman Romero-Ortuno

**Affiliations:** 1 Central Catalonia Chronicity Research Group (C3RG), Centre for Health and Social Care Research (CESS), Faculty of Medicine, University of Vic-Central University of Catalonia (UVIC-UCC), Rambla Hospital 52, 08500 Vic, Barcelona, Spain; 2 Geriatric and Palliative Care Department, Hospital Universitari de La Santa Creu and Hospital Universitari de Vic. Vic, Barcelona, Spain; 3 Chronic Care Program, Ministry of Health, Generalitat de Catalunya, Catalonia, Spain; 4 Data Analysis and Modelling Research Group, Department of Economics and Business, University of Vic-Central University of Catalonia (UVIC-UCC), Barcelona, Spain; 5 Discipline of Medical Gerontology, School of Medicine, Trinity College Dublin, Dublin, Ireland; 6 Mercer’s Institute for Successful Ageing, St James’s Hospital, Dublin, Ireland; 7 Global Brain Health Institute, Trinity College Dublin, Dublin, Ireland

**Keywords:** Frailty, Frailty transitions, Intermediate care, Geriatrics, Older people

## Abstract

**Background:**

Frailty is a dynamic condition that is clinically expected to change in older individuals during and around admission to an intermediate care (IC) facility. We aimed to characterize transitions between degrees of frailty before, during, and after admission to IC and assess the impact of these transitions on health outcomes.

**Methods:**

Multicentre observational prospective study in IC facilities in Catalonia (North-east Spain). The analysis included all individuals aged ≥ 75 years (or younger with chronic complex or advanced diseases) admitted to an IC facility. The primary outcome was frailty, measured by the Frail-VIG index and categorized into four degrees: no frailty, and mild, moderate, and advanced frailty. The Frail-VIG index was measured at baseline (i.e., 30 days before IC admission) (Frail-VIG_0_), on IC admission (Frail-VIG_1_), at discharge (Frail-VIG_2_), and 30 days post-discharge (Frail-VIG_3_).

**Results:**

The study included 483 patients with a mean (SD) age of 81.3 (10.2) years. At the time of admission, 27 (5.6%) had no frailty, and 116 (24%), 161 (33.3%), and 179 (37.1%) mild, moderate, and severe frailty, respectively. Most frailty transitions occurred within the 30 days following admission to IC, particularly among patients with moderate frailty on admission. Most patients maintained their frailty status after discharge. Overall, 135 (28%) patients died during IC stay. Frailty, measured either at baseline or admission, was significantly associated with mortality, although it showed a stronger contribution when measured on admission (HR 1.16; 95%CI 1.10–1.22; *p* < 0.001) compared to baseline (HR 1.10; 1.05–1.15; *p* < 0.001). When including frailty measurements at the two time points (i.e., baseline and IC admission) in a multivariate model, frailty measured on IC admission but not at baseline significantly contributed to explaining mortality during IC stay.

**Conclusions:**

Frailty status varied before and during admission to IC. Of the serial frailty measures we collected, frailty on IC admission was the strongest predictor of mortality. Results from this observational study suggest that routine frailty measurement on IC admission could aid clinical management decisions.

**Supplementary Information:**

The online version contains supplementary material available at 10.1186/s12877-022-03378-9.

## Background

Frailty is commonly defined as dysregulation in multiple physiological systems accompanied by increased vulnerability to stressors, and it negatively influences both health outcomes (e.g., mortality) and the use of resources (e.g., hospitalization) [1]. The prevalence of frailty among community-dwelling individuals aged over 65 is estimated at around 10% [1]. However, nearly half of older adults admitted to acute hospitals are frail [2, 3], a percentage that can increase up to 85% in intermediate care (IC) hospitals and long-term care facilities [4].

Studies investigating frailty in the hospital setting suggest a two-way relationship between frailty and hospitalization. On one side, frail older people have twice the risk of being hospitalized compared to robust older people [5]. On the other hand, hospitalization often results in worsening of the frailty status and is associated with poorer hospital outcomes, including in-hospital and 30-day post-admission mortality [5–7]. In this relationship, frailty must be understood as a dynamic condition, with people transitioning between states of no frailty, pre-frailty, and different degrees of frailty [1, 8, 9]. These transitions are usually precipitated by intercurrent processes, often leading to hospitalization [7]. However, many different tools for measuring frailty have been proposed, with remarkable differences in their capacity to predict relevant health outcomes between tools, even when used in the same cohort [10], thus posing comparability challenges across studies [11].

The dynamic nature of age-related conditions, such as frailty, stresses the need for conducting longitudinal research to better understand frailty fluctuations and trajectories over a life span [1, 12, 13]. To date, most longitudinal analyses of frailty cover large time intervals (e.g., over years) in community-dwelling older people [7, 14–17], provide an epidemiological (rather than clinical) approach from health information systems data [14, 15, 18], or assess transitions between states of no frailty and frailty [15, 16]. However, no studies have prospectively evaluated transitions between different levels of frailty in the IC setting using specific instruments routinely used in regular practice. Therefore, we aimed to measure frailty-degree transitions in older people hospitalized at IC facilities through the serial application of a frailty index and assess the impact of these transitions on health outcomes.

## Methods

### Study design and participants

This was a prospective observational study conducted at the Geriatrics and Palliative Care Department of Osona (Barcelona, Spain) between July 2018 and September 2019. This department provides care to a catchment population of 156,000 inhabitants through a domiciliary care service (hospital at home unit) and two IC hospitals (University Hospital of Santa Creu [Vic, Spain], and Hospital Sant Jaume [Manlleu, Spain]; 170 hospital beds in total), which include a palliative care unit, a rehabilitation unit, a psychogeriatric unit, and a mixed unit. Approximately half of the patients are transferred from the reference acute hospital, and the other half comes from regional hospital emergency rooms and/or from home upon the general practitioner’s request.

All individuals aged ≥ 75 years admitted to the IC facilities within the study period were consecutively offered to enrol in the study. Younger patients were also included if they met the criteria of chronic complex patients (PCC, *Pacient Crònic Complex*), advanced chronic disease (MACA, *Malaltia Crònica Avançada*), according to Catalan Health Department criteria [19]. Individuals who could not have been followed up at home 30 days post-IC discharge for geographical reasons were excluded from the study.

The study protocol was approved by the Ethics Committee of the University Hospital of Vic (2,018,958/PR189), and all participants provided written informed consent to participate in the study. The study results are reported according to the Strengthening the Reporting of Observational Studies in Epidemiology (STROBE) guidelines [20].

### Variables and data sources

The primary outcome was the change between different degrees of frailty as assessed with a frailty index, which is a sensitive tool for measuring frailty change with good ability to predict mortality and other adverse health outcomes [21–23]. For this study, we chose the Frail-VIG index, which consists of 22 dichotomous questions that allow identifying 25 deficits from various domains (including socioeconomic status, estimated on the basis of the social history and/or other relevant available information) and has been shown to be a reliable, feasible, and valid tool to assess the degree of frailty in hospitalized older people [24]. The Frail-VIG index also has a good discriminative capacity for the degree of frailty and a high predictive ability for mortality [25–28].

The degree of frailty was assessed at four time points: baseline status at 30 days before admission to the IC facility (Frail-VIG_0_), within the first 48 h after admission (Frail-VIG_1_), at discharge (Frail-VIG_2_), and at 30 days post-discharge (Frail-VIG_3_). Frail-VIG scores were obtained by hospital health professionals (doctors and nurses), who were already trained in the use of the Frail-VIG index as it is used in routine clinical practice in the local setting. Frail-VIG_0_ was retrospectively assessed by anamnesis of the patient and/or main caregiver, who reported on the patient’s status approximately one month before IC admission. The Frail-VIG_3_ score was obtained by research nurses during home visits. Two of the original Frail-VIG index items were tailored to the study by removing references to temporality (the final version and changes introduced to the Frail-VIG questionnaire are provided in the Supplementary Appendix).

For the comparative analysis between frailty groups at baseline, patients were grouped into four categories, as described elsewhere: [26, 27] no frailty (Frail-VIG index score < 0.2); mild frailty (Frail-VIG index score 0.2–0.35); moderate frailty (Frail-VIG index score 0.36–0.5), and advanced frailty (Frail-VIG index score > 0.5).

Sociodemographic variables included age, sex, and current place of residence. Clinical variables included diagnoses and clinical conditions on IC admission, assessed according to the items of the Frail-VIG index [24, 25] as described in the Supplementary Appendix, date of discharge or death, and all variables included within the Frail-VIG index. We also collected resource use information, including the IC admission unit (i.e., palliative care unit, rehabilitation unit, psychogeriatric unit, mixed unit, and domiciliary care unit), and the length of stay in IC. The instrumental activities of daily living (IADL) were selected from the Lawton-Brody scale considering the items with lower risk of gender bias, as described previously [25].

### Statistical analysis

Categorical variables were described as frequency and percentage over available data, whereas quantitative variables were described as the mean and the standard deviation (SD), without imputing missing data. The association between clinical and demographic characteristics of patients in each frailty degree were assessed using the chi-square test for categorical variables and t-test for quantitative and categorical variables. The transition probabilities between the different levels of frailty/death corresponded to the proportion of patients experiencing a given transition. Finally, the relationship between frailty and the risk of mortality was assessed with a Cox proportional hazards model using the frailty measure at baseline and IC admission. For all hypothesis tests, the significance threshold was set at a two-sided alpha value of 0.05. Descriptive analyses and comparisons regarding demographic and clinical variables were computed using SPSS (Version 28.0. Armonk, NY: IBM Corp.), whereas survival analyses were performed using the Survival and msSurv (multi-state models) packages from the R project [29]. Transitions between frailty levels were plotted using gplot2 and ggalluvial packages from the R project.

## Results

### Patient characteristics

The study included 483 patients: 398 (82.4%) aged ≥ 75 years and 65 (13.5%) aged < 75 years and meeting PCC or MACA criteria, all admitted to the Geriatrics and Palliative Care Department IC facilities. Table 1 summarizes the main demographic and clinical characteristics of the included patients, according to the baseline (i.e., one month before IC admission) frailty status. All clinical characteristics significantly varied according to the baseline frailty status; however, significant differences were not observed in demographic characteristics such as age and usual residence.

**Table 1:** Baseline characteristics of the study cohort at Frail-VIG0 time point (i.e., one month before admission)

**Variable**	**All** *n* = 483	**Frailty status **^**a**^				*p*-value
		**No ** **frailty ** *n* = 115	**Mild** **frailty ** *n* = 187	**Intermediate frailty ** *n* = 122	**Advanced frailty ** *n* = 59	
Age, mean (SD)	81.28(10.21)	79.97(9.22)	80.74(10.90)	82.46(10.58)	83.06(8.63)	0.120
Sex, women (%)	260(53.80)	64(55.65)	101(54.01)	64(52.46)	31(52.54)	0.962
Usual habitat(%)						
with family	317(65.63)	74(64.34)	120(64.17)	86(70.49)	37(62.71)	0.623
with caregiver	23(4.76)	1(0.86)	7(3.74)	8(6.56)	7(3.74)	0.008
Alone	116(24.02)	40(34.78)	56(29.95)	16(13.11)	4(6.77)	< 0.001
Nursing Home	27(5.59)	0(0.00)	4(2.14)	12(9.83)	11(18.64)	< 0.001
Unit of income (%)						
Palliative care unit	190(39.34)	40(34.78)	83(44.39)	42(34.43)	25(42.37)	0.216
Rehabilitation unit	113(23.40)	40(34.78)	40(21.39)	25(20.49)	8(13.56)	0.006
Psicogeriatric unit	79(16.36)	6(5.21)	23(12.30)	35(28.69)	15(25.42)	< 0.001
Hospital-at-home unit	49(10.14)	23(20.00)	20(10.70)	5(4.10)	1(1.69)	< 0.001
HSJ	52(10.76)	6(5.22)	21(11.23)	15(12.30)	10(16.95)	0.095
Stay average. Median (IQR)	20(25)	21(26)	21(28)	21(25)	14(20)	0.113
Individual variables^b^						
IADLs (0–3), mean (SD)	1.35 (1.28)	0.17 (0.46)	1.00 (1.10)	2.36 (0.88)	2.76 (0.54)	< 0.001
Barthel index (0–100), mean (SD)	76.84(25.64)	91.31(17.93)	86.03(15.86)	64.19(25.54)	42.26(25.23)	< 0.001
Malnutrition (%)	135(27.85)	17(14.78)	47(25.13)	39(31.98)	33(55.93)	< 0.001
Cognitive impairment (%)	158(32.71)	2(1.74)	41(221.93)	65(53.28)	50(84.75)	< 0.001
Depressive syndrome (%)	139(28.78)	11(9.57)	44(23.53)	53(43.44)	31(52.54)	< 0.001
Insomnia/anxiety (%)	225(46.58)	24(20.87)	85(45.45)	76(62.30)	40(67.80)	< 0.001
Social vulnerability (%)	74(15.32)	4(3.48)	29(15.51)	23(18.85)	18(30.51)	< 0.001
Delirium (%)	67(13.87)	0(0.00)	10(5.35)	29(23.77)	28(47.46)	< 0.001
Falls (%)	99(20.49)	6(6.06)	39(39.39)	37(37.37)	17(17.18)	0.004
Ulcers (%)	51(10.55)	6(5.22)	16(8.56)	16(13.11)	13(22.03)	0.004
Polypharmacy (%)	389(80.54)	68(59.13)	160(85.56)	104(85.25)	57(96.61)	< 0.001
Dysphagia (%)	76(15.73)	2(1.74)	16(8.56)	20(16.39)	38(64.41)	< 0.001
Pain (%)	117(24.22)	8(6.96)	58(31.02)	32(26.23)	19(32.20)	< 0.001
Dyspnea (%)	46(9.52)	1(0.87)	15(8.02)	19(15.57)	11(18.64)	< 0.001
Cancer (%)	121(25.05)	24(20.87)	60(32.08)	23(18.85)	14(23.73)	0.036
Chronic respiratory disease(%)	110(22.77)	22(19.13)	41(21.93)	36(29.51)	11(18.64)	0.198
Chronic Cardiac disease (%)	217(44.92)	34(29.57)	75(40.11)	70(57.38)	38(64.41)	< 0.001
Chronic Neurological disease (%)	65(13.46)	7(6.09)	20(10.70)	21(17.21)	17(28.81)	< 0.001
Chronic Digestive disease (%)	36(7.45)	2(1.74)	18(9.63)	8(6.56)	8(13.56)	0.018
Chronic Renal disease (%)	189(39.13)	25(21.74)	70(37.43)	56(45.90)	38(64.41)	< 0.001

^a^No frailty (Frail-VIG index score < 0.2), mild frailty (Frail-VIG index score 0.2–0.35), moderate frailty (Frail-VIG index score 0.36–0.5), and advanced frailty (Frail-VIG index score > 0.5)

^b^Criteria for clinical conditions are defined in the Supplementary Appendix

*IADLs* Instrumental of activities of daily living (adapted from the Lawto-Brody scale, as described previously) [25]. *IQR* Interquartile range (25^th^ and 75^th^ percentiles), *SD* Standard deviation

The mean (SD) length of IC stay in the overall sample was 20 (25) days, with significant differences between IC units: 20 (16) for palliative care, 38 (27) for rehabilitation, 24 (15) for psychogeriatric, 36 (31) for mixed, and 8 (3) for domiciliary care unit (*p* < 0.001). Twenty-seven (5.6%) patients were lost to follow-up after IC discharge and, therefore, lacked data for the post-discharge assessment; the frailty degree of these patients at discharge was as follows: 7 no frail, 11 mild frailty, 3 moderate frailty, and 6 advanced frailty.

### Frailty transitions

**Fig. 1:**
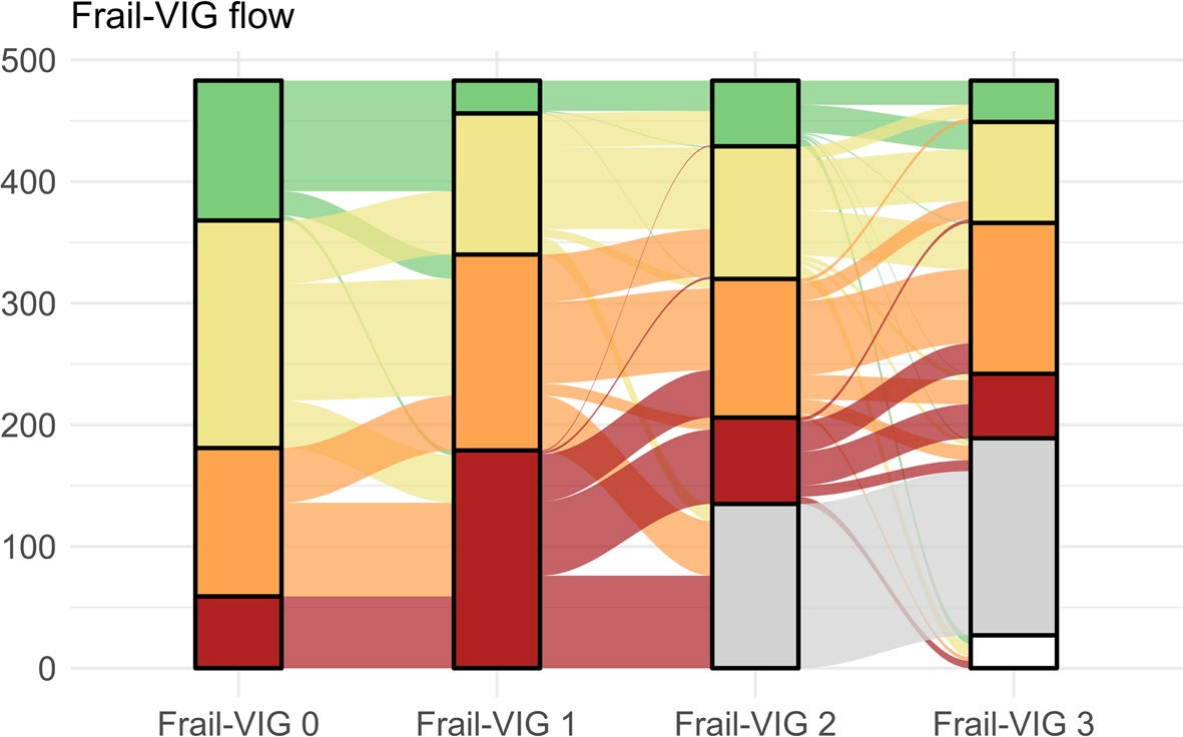
Distribution of the study cohort across frailty status categories at each assessment point: one month before admission (Frail-VIG_0_), within the first 48 h after admission (Frail-VIG_1_), at discharge (Frail-VIG_2_), and at 30-day post-discharge (Frail-VIG_3_). Green: No frailty (Frail-VIG index score < 0.2). Yellow: mild frailty (Frail-VIG index score 0.2–0.35). Orange: moderate frailty (Frail-VIG index score 0.36–0.5). Red: advanced frailty (Frail-VIG index score > 0.5). Grey: death, White: missing values

Figure 1 summarizes the patients’ distribution across frailty severity levels in each of the assessments, and the transitions that occurred between these time points. Between the baseline assessment and IC admission, all patients either maintained or worsened their frailty severity. Conversely, a remarkable number of patients improved their frailty status between IC admission and discharge; most improvements occurred to the next-lower frailty degree. The number of improvements within the post-discharge period was more moderate. Overall, most of the transitions to death occurred from the advanced and moderate frailty levels. The mortality proportion in each sub-type of IC unit was 90% in palliative care, 11% rehabilitation, 15% psychogeriatric, 19% mixed, and 0% in domiciliary care.

**Fig. 2:**
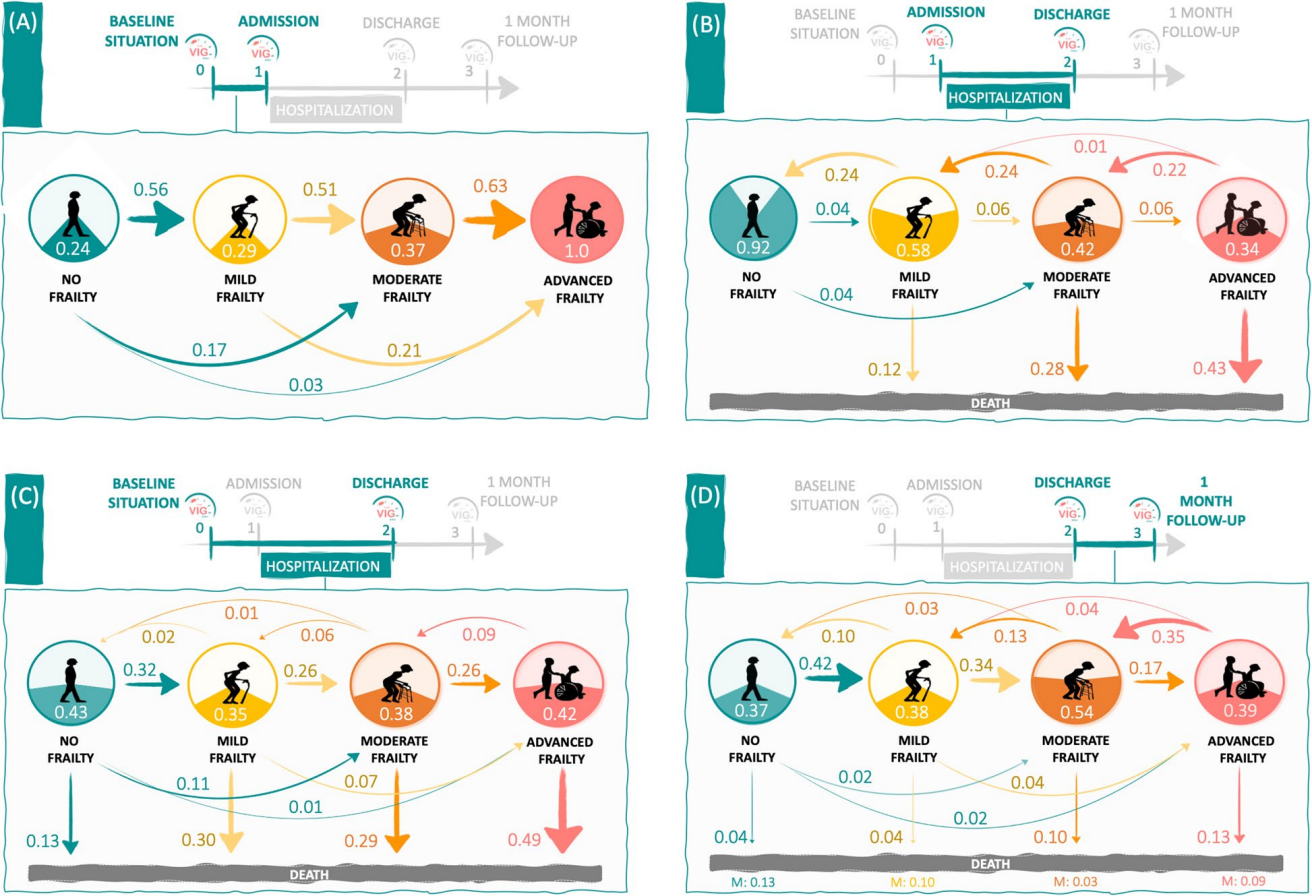
Transition probabilities between frailty statuses in all stages: between baseline (i.e., one month before admission) and admission (**A**), between admission and discharge (**B**), between baseline and discharge (**C**), between discharge and 30-day post-discharge follow-up (**D**). No frailty (Frail-VIG index score < 0.2), mild frailty (Frail-VIG index score 0.2–0.35), moderate frailty (Frail-VIG index score 0.36–0.5), and advanced frailty (Frail-VIG index score > 0.5). The arrow size is proportional to the transition probability. The probability of remaining in the same status for a given period is displayed within the circle. M: missing values

The transition probabilities derived from the assessment result at each time point are depicted in Fig. 2. Compared to the baseline status (Frail-VIG_0_), most patients had worsened their frailty at the time of IC admission (Frail-VIG_1_), more likely by increasing one degree in the 4-state scale. The probability of worsening between baseline and IC admission was highest in patients with moderate frailty (0.63); regardless of the baseline status, patients had higher probability to increase one frailty degree (0.56, 0.51, and 0.63) than remaining in the non-frail to moderate frailty status (0.24, 0.29, and 0.37) or increasing by two frailty degrees their frailty status (0.17, 0.21, and 0.03) (Fig. 2A). Conversely, following admission, the probability of remaining in the same status (0.92, 0.58, 0.42, and 0.34) or improving it by one frailty degree (0.22 and 0.24) increased inversely with frailty on admission, and the probability of dying (0.12, 0.28, and 0.43) increased with the worsening of frailty status (Fig. 2B). Individuals with moderate frailty on admission who did not maintain their frailty status at discharge more frequently transitioned towards death (0.28) or improvement to mild frailty (0.24), but rarely worsened to advanced frailty (0.06). Taken together, the transition probabilities between the entire period lasting from baseline (Frail-VIG_0_) to IC discharge (Frail-VIG_2_) showed that patients had higher probability of recovering the baseline state (0.35 – 0.43) or worsening by one frailty degree (0.32 and 0.26) than improving their frailty degree (< 0.10) (Fig. 2C). During this period, patients with mild or moderate frailty at baseline had similar probability to worsen their frailty status (0.26) and dying (0.30 and 0.29). The probability of dying was highest among patients with advanced frailty at baseline, although nearly half of them (42%) were discharged with the same status as baseline. Finally, during the 30-day post-discharge follow-up, frailty remained dynamic. Although patients were more likely to remain in the same status, one third of patients without frailty or with mild frailty at discharge worsened during this period, and one third of patients with advanced frailty improved. Considering the entire investigated period, most of the transitions towards higher frailty occurred during the pre-admission stage; the IC admission period accounted for most of the deaths.

**Fig. 3:**
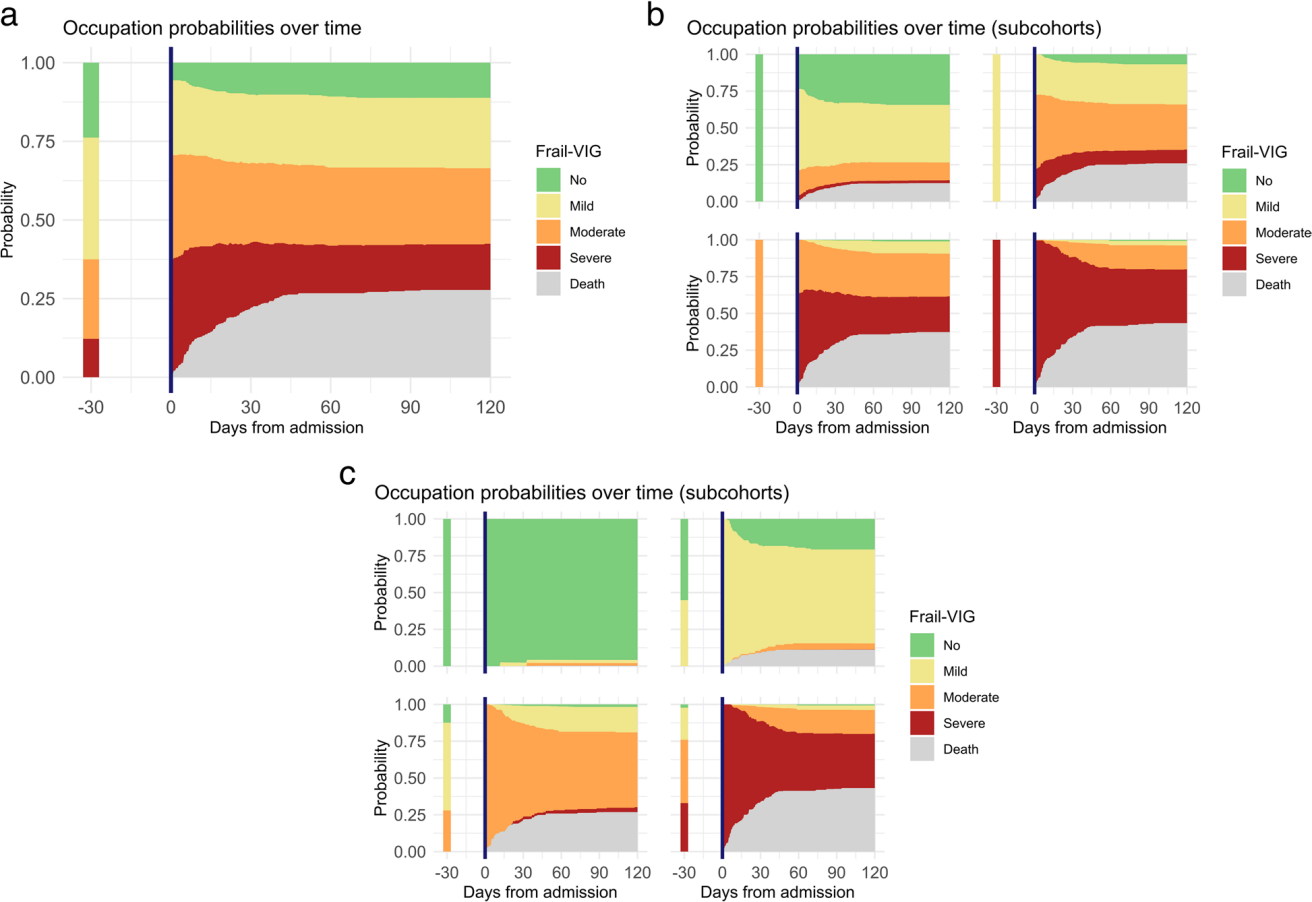
Occupation probabilities for each frailty status, estimated using a survival analysis of frailty status between admission to the intermediate care facility and discharge **A** Entire cohort. **B** stratified by frailty status at baseline. **C** stratified by frailty on admission

Another aspect to consider in the dynamic picture of frailty is the length of IC stay, which varied among patients in the cohort. Figure 3 shows the results of the multistate model analysis, which provides a probability of a given frailty status at a specified time point between IC admission and discharge. The analysis revealed that most transitions occurred within the first 30 days after IC admission, except for patients with advanced frailty at baseline, who needed more time to reach a more stable state (Fig. 3A). This trend of the overall cohort was particularly prominent in patients with moderate frailty at baseline (Fig. 3B).

### Frailty and survival

**Fig. 4:**
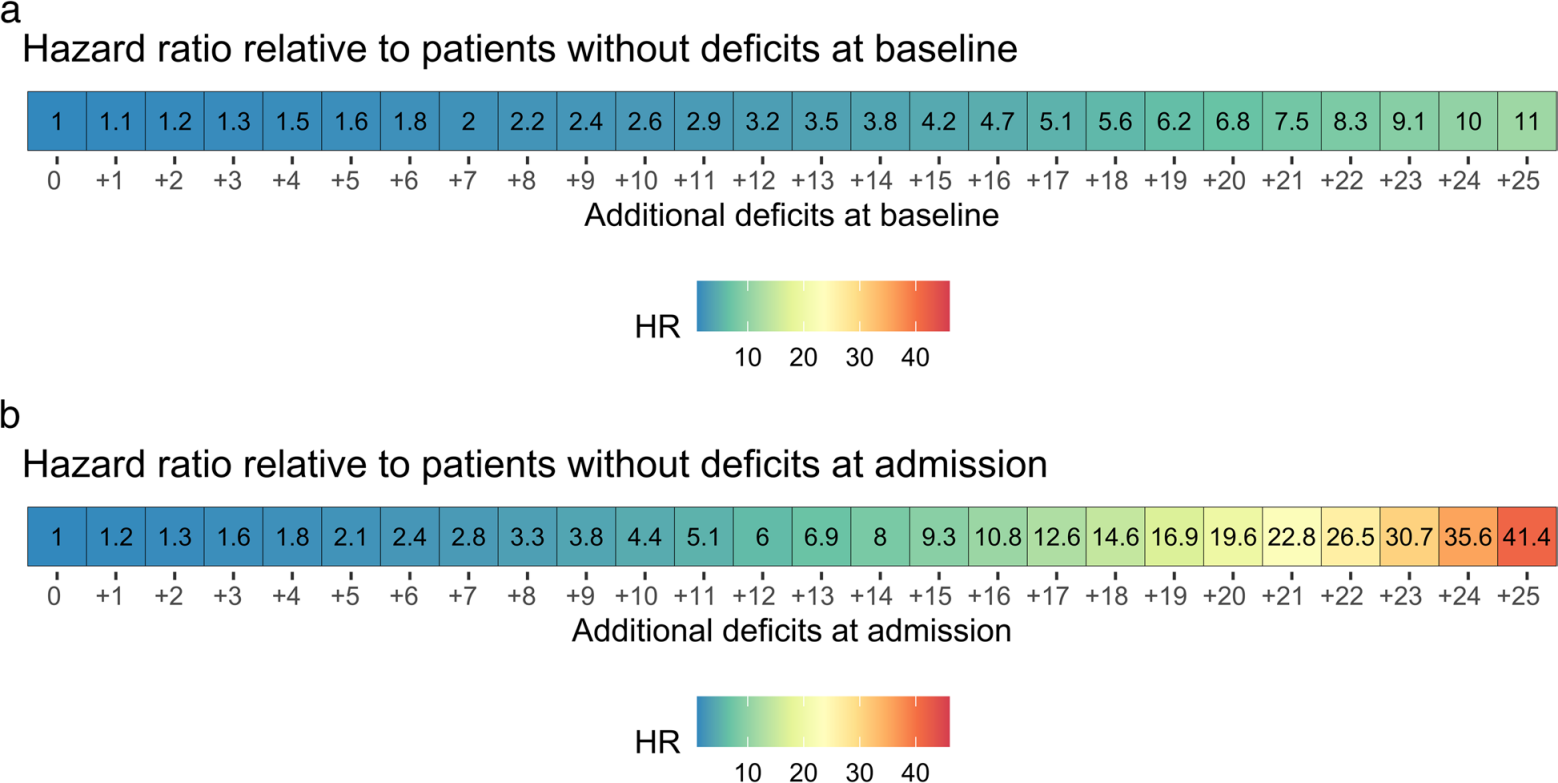
Increase in mortality risk (hazard ratio) associated with each additional deficit in the Frail-VIG scale. **A** frailty status at baseline (i.e., one month before admission); the HR associated with a gain of one additional deficit at baseline was 1.10 (95% CI 1.05 – 1.15), *p* < 0.001. **B** frailty status on admission; the HR associated with a gain of one additional deficit at baseline was 1.16 (1.10 – 1.22), *p* < 0.001

Overall, 135 (28%) patients died during the IC stay. The frailty status, measured either at baseline or on IC admission, was associated with mortality; however, it showed a stronger contribution to mortality when measured on admission (HR 1.16; 95% CI 1.10 – 1.22; *p* < 0.001) compared to baseline (HR 1.10; 1.05 – 1.15; *p* < 0.001). Figure 4 shows the increased risk associated with each additional deficit in the 22-item Frail-VIG scale that identified 25 deficits at baseline and IC admission. When including the baseline (Frail-VIG_0_) and admission (Frail-VIG_1_) measures in a multivariate model, Frail-VIG_0_ did not show a significant contribution to the model: HRs for Frail-VIG_0_ and Frail-VIG_1_ in the multivariate model were 0.99 (95% CI 0.94 – 1.06; *p* = 0.851) and 1.17 (1.09 – 1.25; *p* < 0.001), respectively.

## Discussion

Our prospective analysis of frailty transitions of older people admitted to IC facilities highlights the complexity of transitions between frailty status in this setting. In our cohort, most transitions occurred within 30 days after IC admission, particularly among patients with moderate frailty on IC admission. These patients more frequently maintained their status during IC stay and had a similar probability of dying and improving to mild frailty, but they rarely transitioned towards advanced frailty within this period. Finally, we found that the degree of frailty, measured using the validated Frail-VIG tool was associated with increased mortality in a dose-dependent way; however, frailty status on IC admission had a much higher predictive value.

A recent systematic review of frailty trajectories by Welstead et al. did not find longitudinal studies conducted in settings other than community-based populations [30]. These studies, which included community-dwelling individuals, typically employed follow-up periods of various years. Some of the studies investigating frailty trajectories in community-dwelling individuals have specifically addressed the question as to whether a point measurement or a change assessment better predicts health outcomes in frail people [14, 31, 32]. These studies have drawn inconsistent conclusions supporting the use of time-point measurements [32], frailty changes [14], or any of the two approaches for predicting mortality in the community setting [31]. Among them, Bai et al. found that the impact of accumulating deficits is more determinant in midlife than old age, suggesting that the optimal approach to frailty assessment may vary depending on age [32].

Regardless of the level of consensus on this question, the trends observed in the community setting may not apply to the IC setting, where frailty is expected to change more rapidly and frequently following a previous acute hospitalization [5–7] or a frailty increase in the community necessitating IC admission [33]. In our setting, the routine assessment on IC admission consists of a retrospective administration of a frailty questionnaire to establish the baseline status of the patient (by anamnesis of the patient and/or patient’s relatives). While this approach is useful for establishing goals, our results indicate that the frailty status on IC admission predicts mortality with a significantly higher accuracy than the same measure one month before admission. This finding suggests that administering the Frail-VIG questionnaire on admission (either by a general practitioner, geriatrician or nurse) might be more useful for screening patients and planning interventions in this setting, although the possibility of rapid transitions at this time point should also be considered. Even though the baseline frailty assessment was conducted retrospectively and the assessment of frailty on IC admission could have been confounded by illness acuity [34], our observational findings support the implementation of routine frailty measurement on IC admission as potentially being more useful for care planning purposes than purely relying on baseline frailty information.

The scope of our results is limited to individuals who were admitted to an IC facility and, therefore, survived the pre-admission stage. The regular practice in the study area is to prioritize admission to individuals with functional loss after an acute hospital stay, end-of-life care needs, or for management of complex diseases and/or geriatric syndromes. However, specific care pathways may differ between countries. Our study included a smaller sample size than previous studies investigating the dynamics of frailty. However, most of these studies are retrospective analyses of population-based datasets that lack information on validated questionnaires and do not assess IC patients at multiple time points.

A limitation is that our study population is heterogeneous regarding the type of IC facility. While the inclusion of patients admitted to different IC sub-settings (e.g., rehabilitation, palliative care, psychogeriatric unit) provides an overarching view of frailty transitions in different care pathways (therefore capturing the real-world scenario), mortality proportions may well differ between sub-units, thus increasing heterogeneity and introducing unbalanced biases in the observation of frailty transitions and prediction of mortality. One example of this potential bias is the higher number of patients discharged from the IC with advanced frailty who improved during the 1-month follow-up period. Although disutility experienced during hospital stay might explain the high probability of improvement at home, the relatively limited number discharged with advanced frailty and alive 1-month after discharge, which are likely to be more representative of those without terminal illness and higher rehabilitation potential, precludes drawing strong conclusions in this regard.

## Conclusions

Our results illustrate the dynamic nature of frailty in IC, which may worsen or improve at any stage and should be therefore measured serially. Our results indicate that admission frailty was a stronger predictor of mortality than the baseline measure; hence, routinely measuring frailty on IC admission may be of more practical value for care planning. Finally, clinicians should be aware that frailty transitions after the first 30 days of IC admission are infrequent, and even though our analysis was limited to two time points, this may help plan the appropriate length of IC stay.

## Supplementary Information

**Additional file 1.**

## Data Availability

The datasets used and/or analysed during the current study are available from the corresponding author on reasonable request.

## Abbreviations

HR: Hazard ratio
MACA: Advanced chronic disease (from Catalan, *Malaltia Crònica Avançada*)
PCC: Chronic complex patient (from Catalan*, Pacient Crònic Complex*)
SD: Standard deviation
STROBE: Strengthening the Reporting of Observational Studies in Epidemiology
