# Supplementary material for: Transitions between degrees of multidimensional frailty among older people admitted to intermediate care: a multicentre prospective study
Source: BMC Geriatr. 2022 Sep 1;22:722. doi: 10.1186/s12877-022-03378-9 (PMC9438217; doi:10.1186/s12877-022-03378-9)
Supplement: Supplementary file 1 — Additional file 1. [file 12877_2022_3378_MOESM1_ESM.docx]

**Transitions between degrees of multidimensional frailty among older people admitted to intermediate care: a multicenter prospective study**

Supplementary appendix

| **DOMAIN** | | **VARIABLE** | **DESCRIPTION** | **FRAIL- VIG INDEX** | **COMMENTS** | | |
| --- | --- | --- | --- | --- | --- | --- | --- |
| **Functio**  **nal** | **IADLs** | **Money management** | Needs help managing financial matters (bank, shops, restaurants) | **BASELINE**  (IF-VIG)**_b_** | NO assistance will be considered necessary if (approximately) 1 month prior to admission the patient was able to deal with money to do the daily shopping; autonomously manage basic bank deposit or withdrawal operations, or pay the restaurant bill. | | |
|  |  |  |  | **ADMISSION**  (IF-VIG_0_ ) | The interviewer considers that the patient **would currently need assistance** to manage their own money and/or do basic money operations ─like giving change─ if they were not hospitalized. | | |
|  |  |  |  | **DISCHARGE**  (IF-VIG_1_ ) | The interviewer considers that the patient **will need assistance** to manage the above-mentioned money operations on discharge. | | |
|  |  | **Telephone use** | Needs help using the telephone | **BASELINE**  (IF-VIG)**_b_** | No assistance will be considered necessary if (approximately) 1 month prior to admission the patient was autonomously able to use the phone; and/or correctly dial some close relatives’ numbers, and/or properly answer the phone. | | |
|  |  |  |  | **ADMISSION**  (IF-VIG_0_ ) | THE INTERVIEWER CONSIDERS that the patient is **currently** able to use a phone ─ **or would hypothetically be able to**─ if they were not hospitalized. | | |
|  |  |  |  | **DISCHARGE**  (IF-VIG_1_ ) | The INTERVIEWER CONSIDERS that the patient will need assistance to use a phone on discharge. | | |
|  |  | **Medication management** | Needs assistance in preparing or administering medications | **BASELINE**  (IF-VIG)**_b_** | Assistance will be considered necessary or unnecessary if **(approximately) 1 month prior to admission**:  * The patient got their blister pack medication prepared and they autonomously self-administered it. In this case, assistance will be considered necessary.  * The patient was not taking any kind of medication. In this case, assistance will not be considered necessary. | | |
|  |  |  |  | **ADMISSION**  (IF-VIG_0_ ) | The interviewer considers that the patient would **currently need assistance** to take **their oral medication** (they are not able to self-administer it). | | |
|  |  |  |  | **DISCHARGE**  (IF-VIG_1_ ) | The interviewer considers that the patient **will need assistance** to prepare or self-administer their medication on discharge. | | |
|  | **ADLs** | **Barthel index (BI)** | No dependency (BI ≥ 95)  Mild-moderate ependency  (BI 90-65)  Moderate-severe Dependency (BI 60-25)  Absolute dependency  (BI ≤20) | **BASELINE**  (IF-VIG)**_b_** | It refers to the patient’s baseline BI before the intercurrent process that has led to admission (in general terms, approximately **1 month prior to admission**). | | |
|  |  |  |  | **ADMISSION**  (IF-VIG_0_ ) | It refers to the patient’s BI during the assessment within the **first 48 hours** (approximately) after admission. | | |
|  |  |  |  | **DISCHARGE**  (IF-VIG_1_ ) | It refers to the patient’s IB **on discharge**. | | |
| **Nutritional** | | **Malnutrition** | Weight loss ≥ 5% in the last 6 months | **BASELINE**  (IF-VIG)**_b_** | Has the patient lost ≥ 5% their weight **during the last 6 months?**  * It is not essential to objectify weight loss. It would be enough to report an approximate figure assessed by anamnesis (“how heavy were they 6 months ago and how heavy are they now?”), or by the patient’s or relatives’ subjective perception (“he’s gone down 1 or 2 sizes”, “she’s lost so much weight”, “he’s melted off”, etc.). | | |
|  |  |  |  | **ADMISSION** (IF-VIG_0_ ) | Idem BASELINE FRAIL-VIG INDEX | | |
|  |  |  |  | **DISCHARGE** (IF-VIG_1_ ) | It refers to whether the patient had lost ≥ 5% their weight during the last 6 months and **has not gained** it back after the current admission. | | |
| **Cognitive** | | **Degree of cognitive impairment** | No cognitive impairment  Mild-moderate cognitive impairment (equivalent to GDS ≤5)  Severe-very severe cognitive impairment (equivalent to GDS ≥ 6) | **BASELINE**  (IF-VIG_b_) | * Categorised in 3 sections through Reisberg’s Global Deterioration Scale (GDS) (Annex 3) as: no cognitive impairment (scored 0); mild-moderate cognitive impairment (equivalent to GDS ≤ 5: scored as 1 point), or severe/ extreme cognitive impairment (equivalent to GDS ≥ 6: scored as 2 points). | | |
|  |  |  |  | **ADMISSION** (IF-VIG_0_) |  |  |  |
|  |  |  |  | **DISCHARGE**  (IF-VIG_1_ ) | Depending on the cognitive status on discharge. | | |
| **Emotional** | | **Depressive syndrome** | Need for antidepressive medication | At some point during the study **(FRAIL-VIGb, 0,1),** the patient will need antidepressant medication if diagnosed with depressive syndrome or any mood disorder (e.g., bipolar disorder) that may require specific treatment with SSRIs (fluoxetine, paroxetine, sertraline, citalopram, fluvoxamine, etc.); SNSRIs (venlafaxine, duloxetine, etc.); TADs (amitriptyline, nortriptyline, etc.), or MAOIs/adjuvants (lithium, etc.). | | | |
|  |  | **Insomnia/anxiety** | Frequent need for benzodiazepines or other psychiatric drugs with a sedative effect for insomnia/anxiety | **BASELINE**  (IF-VIG)**_b_** | * The patient will need treatment in case of insomnia or anxiety disorder treated with benzodiazepines *(lorazepam, alprazolam, diazepam, etc.)*; hypnotics *(clomethiazole, zolpidem, etc.)*; sedative antidepressants *(trazodone, mirtazapine, etc.)*, or any other treatment used for this purpose *(sedative antihistamines, e.g., hydroxyzine).* | | |
|  |  |  |  | **ADMISSION**  (IF-VIG_0_ ) | On admission, the patient will need insomnia and/or anxiety disorder treatment *(benzodiazepines, hypnotics, antidepressants or sedative antihistamines).* | | |
|  |  |  |  | **DISCHARGE**  (IF-VIG_1_ ) | On discharge, the patient will need insomnia and/or anxiety disorder treatment *(benzodiazepines, hypnotics, antidepressants or sedative antihistamines).* | | |
| **Social** | | **Social vulnerability** | Do health care professionals perceive the presence of social vulnerability? | **BASELINE**  (IF-VIG_b_) | *Consideration will be given at the discretion of the professionals that assess the patient. Situations such as the following will be considered: patients who live alone under poor hygiene conditions; and/or whose family have a limited support capacity, and/or difficulties to take responsibility for the patient’s care; family conflict relationships, or lack of economic resources.  * In case the patient lives in an institution or elderly nursing home, it will be considered that there is not a social vulnerability situation. | | |
|  |  |  |  | **ADMISSION** (IF-VIG_0_) |  |  |  |
|  |  |  |  | **FRAIL-VIG INDEX_1_** | It refers to the social situation on discharge. | | |
| **Geriatric syndromes** | | **Delirium** | Presence of delirium and/or behaviour disorder requiring neuroleptics in the last 6 months. | **BASELINE**  (IF-VIG)**_b_** | *It will be considered as a deficit if, at some point during **the last 6 months**, the patient has been prescribed typical (haloperidol, etc.), or atypical neuroleptics (risperidone, quetiapine, etc.). This consideration does not include conditional medication that may have not been administered.  In case of a mixed/hypoactive confusional state not requiring treatment, a deficit could be considered if there is a clear diagnosis after using a validated scale (CAM, 4AT, etc.). | | |
|  |  |  |  | **ADMISSION**  (IF-VIG_0_ ) | **On admission**, the patient presents an acute confusional state and/or a behaviour disorder requiring a neuroleptic treatment regimen (whether the state/disorder is the reason for consultation or the diagnosis).  In case of a mixed/hypoactive confusional state not requiring treatment, a deficit could be considered if there is a clear diagnosis after using a validated scale (CAM, 4AT, etc.). | | |
|  |  |  |  | **DISCHARGE**  (IF-VIG_1_ ) | **On discharge**, the patient presents an acute confusional state and/or a behaviour disorder and needs to continue with the neuroleptic treatment regimen.  In case of a mixed/hypoactive confusional state not requiring treatment, a deficit could be considered if there is a clear diagnosis after using a validated scale (CAM, 4AT, etc.). | | |
|  |  | **Falls** | In the last 6 months, ≥2 falls or hospitalization due to a fall. | **BASELINE**  (IF-VIG)**_b_** | * At some point during **the last 6 months**, the patient has taken a fall related to extrinsic factors (tripping over a rug, accidents, etc.) and/or intrinsic ones (syncope, pathological fracture, etc.).  * The concept “hospitalization” includes hospital admission (>24 hours) for any kind of fracture and/or underlying aetiology that may have caused the fall (cardiac arrhythmia, pulmonary thrombosis, etc.). | | |
|  |  |  |  | **ADMISSION**  (IF-VIG_0_ ) | Idem FRAIL-VIG_b_, **adding the current admission** if the reason for consultation was the fall and/or such fall is a consequence of the patient’s medical condition. | | |
|  |  |  |  | **DISCHARGE**  (IF-VIG_1_ ) | Idem FRAIL-VIG_0_. | | |
|  |  | **Ulcers** | Presence of ulcer (pressure or vascular, any grade) | * At some point during the study **(FRAIL-VIGb, 0,1)**, the patient may present some type of ulcer (decubitus, varicose, arterial, tumoral, traumatic, etc.) of any grade (I, II, II, IV). | | | |
|  |  | **Polypharmacy** | Taking ≥ 5 drugs | **BASELINE**  (IF-VIG)**_b_** | It refers to the baseline treatment (before admission):  * Any type of medication, including bronchodilators, collyrium and transdermal patches.  * The combination of active principles in a single tablet (e.g., enalapril + hydrochlorothiazide) needs to be counted as separate medications.  * Conditional medication is not included. | | |
|  |  |  |  | **ADMISSION**  (IF-VIG_0_ ) | On admission, the patient prescribed medication is ≥5, excluding conditional medication. | | |
|  |  |  |  | **DISCHARGE**  (IF-VIG_1_ ) | On discharge, the patient prescribed medication is ≥5, excluding conditional medication. | | |
|  |  | **Dysphagia** | Difficulty swallowing when eating or drinking? Presence of aspiration respiratory infections during the last 6 months? | **BASELINE**  (IF-VIG)**_b_** | It refers to the **baseline situation/the last 6 months prior to admission**:  * Medical diagnosis may be made from anamnesis of the patient and/or family members, and/or using a specific test (volume-viscosity, videofluoroscopy, etc.).  * Dysphagia is not considered present if there is neither suggestive semiology with the usual dysphagia management strategies (thickened fluids, pureed diet, etc.), nor related microaspirations.  * However, in case a nasogastric tube or PEG is required, dysphagia will be considered present. | | |
|  |  |  |  | **ADMISSION**  (IF-VIG_0_ ) | On admission, the patient has dysphagia and requires specific management strategies and/or may have contracted any respiratory infection caused by aspiration during the last 6 months, including the reason for consultation of the current admission. | | |
|  |  |  |  | **DISCHARGE**  (IF-VIG_1_ ) | Dysphagia will be considered present in case dysphagia management strategies are still required on discharge, and/or the patient may have contracted any respiratory infection caused by aspiration during the last 6 months, including the current admission. | | |
| **Severe symptoms** | | **Pain** | Need for ≥ 2 conventional analgesics and/or strong opioids for pain control | **BASELINE**  (IF-VIG)**_b_** | It refers to the baseline treatment (before admission) including:  * Conventional analgesics (paracetamol, any NSAID, dipyrone, etc.) and weak opioids (tramadol, codeine, etc.).  * Strong opioids (morphine, fentanyl, oxycodone, methadone, hydromorphone, tapentadol, etc.). | | |
|  |  |  |  | **ADMISSION**  (IF-VIG_0_ ) | On admission, the patient requires ≥2 conventional analgesics and/or strong opioids to control pain, including pain caused by the reason for consultation on admission. | | |
|  |  |  |  | **DISCHARGE**  (IF-VIG_1_ ) | On discharge, the patient requires ≥2 conventional analgesics and/or strong opioids to control pain. | | |
|  |  | **Dyspnea** | Basal dyspnea impeding the ability to leave the house and/or opioids are frequently needed | **BASELINE**  (IF-VIG)**_b_** | The patient presents **baseline dyspnoea at rest/during small physical efforts**, which limits their personal autonomy (to the extent of having to be locked down home). Patients would require full assistance to go on the street (using a transport wheelchair).  * Strong opioids (morphine, fentanyl, etc.) are specifically prescribed to control dyspnoea. | | |
|  |  |  |  | **ADMISSION**  (IF-VIG_0_ ) | **On admission**, the patient presents dyspnoea at rest/during small physical efforts, and/or requires opioids for symptomatic control. | | |
|  |  |  |  | **DISCHARGE**  (IF-VIG_1_ ) | **On discharge**, the patient presents dyspnoea at rest or during small physical efforts, and/or requires opioids for symptomatic control. | | |
| **Diseases (+)** | | **Cancer** | Active cancer | * It includes any type of cancer: solid tumour cancer, haematological cancer or skin cancer.  * It does not include either premalignant lesions (villous adenoma, epithelial dysplasia, etc.), or cured oncological diseases/showing no recidivism. | | | |
|  |  | **Respiratory** | Presence of any type of chronic respiratory disease *(COPD, restrictive lung disease...)* | * It includes any type of chronic respiratory disease, either obstructive lung disease (chronic bronchitis, pulmonary emphysema, chronic asthma, etc.); restrictive lung or extra-pulmonary disease (pulmonary fibrosis, severe kyphosis, etc.), or mixed lung disease. | | | |
|  |  | **Cardiac** | Presence of any type of chronic heart disease *(heart failure, ischemic cardiopathy, arrhythmia)* | * It includes any type of chronic heart disease, either heart failure (systolic or diastolic), ischemic heart disease or heart arrhythmia (auricular fibrillation, flutter, AV block, etc.). | | | |
|  |  | **Neurological** | Presence of any type of neurodegenerative disease *(Parkinson, ALS,...)* or a history of stroke *(ischemic or hemorrhagic).* | * It includes any type of neurodegenerative disease (Parkinson’s disease, ALS, PSP, etc.). However, it does not include neurodegenerative dementias (already assessed in the “Dementia” section).  * It includes any type of stroke history (regardless the level and origin). | | | |
|  |  | **Digestive** | Presence of any type of chronic digestive disease *(chronic liver disease, cirrhosis, chronic pancreatitis, inflammatory bowel disease,…)* | * It includes any type of chronic digestive or liver disease. | | | |
|  |  | **Renal** | Presence of chronic renal failure *(GFR <60)* | *The patient is considered to present chronic renal failure if the GFR is <60 in the baseline situation (situations of acute kidney failure are excluded), and/or needs chronic kidney replacement therapy (hemodialysis or peritoneal dialysis). | | | |
| **Frail-VIG index =** | | | | | | 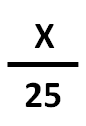 |  |

Table S1: Description and characteristics of the Frail-VIG index. IF-VIG_b:_ Baselina Frail-VIG Index. IF-VIG_0_: Frail-VIG Index on admission. IF-VIG_1_: Frail VIG Index on discharge. ADLs: Basic Activities of Daily Living. IAVDs: Instrumental Activities of Daily Living. BI: Barthel Index. ALS: amyotrophic lateral sclerosis. COPD: Chronic Obstructive Pulmonary Disease. GFR: Glomerular Filtration Rate. GDS: Global Deterioration Scale. (+) **Two point are scored if the patient presents criteria for advanced chronic illness on the NECPAL test** (Annex 2; available at: http://ico.gencat.cat/web/.content/minisite/ico/professionals/documents/qualy/arxius/NECPAL-3.0-ENGLISH_full-version.pdf).
